# Ring opening polymerisation of ɛ-caprolactone with novel microwave magnetic heating and cyto-compatible catalyst

**DOI:** 10.3389/fbioe.2023.1123477

**Published:** 2023-02-13

**Authors:** Kaiyang Wang, Ming Ni, Adam A. Dundas, Georgios Dimitrakis, Derek J. Irvine

**Affiliations:** ^1^ Shanghai Engineering Technology Research Center for Pharmaceutical Intelligent Equipment, Shanghai Frontiers Science Center for Druggability of Cardiovascular Non-Coding RNA, Institute for Frontier Medical Technology, Shanghai University of Engineering Science, Shanghai, China; ^2^ Department of Orthopaedics, Shanghai Key Laboratory for Prevention and Treatment of Bone and Joint Diseases, Shanghai Institute of Traumatology and Orthopaedics, Ruijin Hospital, Shanghai Jiao Tong University School of Medicine, Shanghai, China; ^3^ Centre for Additive Manufacturing, Faculty of Engineering, University of Nottingham, Nottingham, United Kingdom; ^4^ George Green Institute for Electromagnetics Research, Faculty of Engineering, University of Nottingham, Nottingham, United Kingdom

**Keywords:** microwave synthesis, magnetic susceptible catalyst, biomaterial fabrication, PCL synthesis, controlled polymerization

## Abstract

We report on the ring-opening polymerization of ɛ-caprolactone incorporated with a magnetic susceptible catalyst, FeCl_3_, *via* the use of microwave magnetic heating (HH) which primarily heats the bulk with a magnetic field (H-field) from an electromagnetic field (EMF). Such a process was compared to more commonly used heating methods, such as conventional heating (CH), i.e., oil bath, and microwave electric heating (EH), which is also referred to as microwave heating that primarily heats the bulk with an electric field (E-field). We identified that the catalyst is susceptible to both the E-field and H-field heating, and promoted the heating of the bulk. Which, we noticed such promotion was a lot more significant in the HH heating experiment. Further investigating the impact of such observed effects in the ROP of ɛ-caprolactone, we found that the HH experiments showed a more significant improvement in both the product Mwt and yield as the input power increased. However, when the catalyst concentration was reduced from 400:1 to 1600:1 (Monomer:Catalyst molar ratio), the observed differentiation in the Mwt and yield between the EH and the HH heating methods diminished, which we hypothesized to be due to the limited species available that were susceptible to microwave magnetic heating. But comparable product results between the HH and EH heating methods suggest that the HH heating method along with a magnetic susceptible catalyst could be an alternative solution to overcome the penetration depth problem associated with the EH heating methods. The cytotoxicity of the produced polymer was investigated to identify its potential application as biomaterials.

## 1 Introduction

In recent years, the development of polymers that are environmentally friendly and biodegradable has generated substantial effort toward the polymerization of cyclic esters (Bartnikowski et al., 2019; Tsang et al., 2019; Li et al., 2021; Lu et al., 2022a; Cao et al., 2023). Poly lactones possess good biodegradability and biocompatibility and have shown great potential due to their mechanical compatibility and ability to mix with other polymers (Fortelny et al., 2019; Tabasum et al., 2019; Yang et al., 2021; Backes et al., 2022). In particular, poly ε-caprolactone (PCL) has been utilized in different fields such as tissue engineering, drug delivery systems, microelectronics, and environmentally friendly packaging (Siddiqui et al., 2018; Zhang et al., 2019; Thakur et al., 2021; Backes et al., 2022; Lu et al., 2022b). It is one of the most important and widely studied poly-lactones thanks to its controllable polymerization characterization (Labet and Thielemans, 2009; Nuyken and Pask, 2013) and biodegradability (Bartnikowski et al., 2019; da Silva and de Torresi, 2019; Suzuki et al., 2021). This has led to numerous research including its catalytic systems, polymerization mechanisms, and processing techniques.

In most commercialized PCL synthesis, ring-opening polymerization (ROP) is the preferable route (Li et al., 2020; Rosa et al., 2021). This process is a one-step polymerization that can be catalysed by various metal complexes ranging from simple metal halides to complex organometallics (Kundys et al., 2018; De Hoe et al., 2022), and can be controlled in terms of the molecular weight (Mwt) and the polydispersity (Ð).

However, the difficulty of removing the catalyst residue and the cytotoxicity associated with such residues in the final product is the major problem that hinders PCL from biomedical applications (Gowda and Chakraborty, 2009; Hege and Schiller, 2014). To decrease the toxicity and improve the energy efficiency towards the principle of green chemistry, various catalyst systems, and processing techniques were studied. Among different catalysts that have been investigated, iron (III) halides were found to be an effective catalyst and can be used in fabricating biocompatible materials (Petrenko et al., 2011; Hege and Schiller, 2014). These studies have paved the way for intensifying the process. However, these reactions were found to be finished in hours even days, making the processing time-consuming and unflavoured for high product throughput (Engel et al., 2019; Dabbaghi et al., 2021).

Microwave heating (MWH) is a processing technique that delivers a fast polymerization rate and shortened reaction time from hours to minutes. Previous studies found that the reaction time could be reduced significantly when utilizing the MWH to the ROP of ɛ-caprolactone, compared to using the conventional heating (CH) method in identical conditions (Liao et al., 2002; Yu et al., 2003; Li et al., 2007; Nguyen et al., 2014; Fimberger and Wiesbrock, 2016). However, the majority of the studies focused on the dielectric materials and their interactions with the electric field (E-field), largely ignoring the presence of the magnetic field (H-field) in a microwave electromagnetic field (EMF) (Tanaka et al., 2008; Horikoshi et al., 2012). Some pioneer studies successfully applied the microwave H-field heating to superconducting materials, magnetic solids, ferrofluids, and aqueous electrolyte solutions (Ceylan et al., 2011; Horikoshi et al., 2012; Borsari et al., 2018; Loharkar et al., 2019; Siebert et al., 2019; Xiong et al., 2021; Chen et al., 2022). However, there is no research on using the microwave H-field heating method for polymerization chemistry.

The purpose of this work was to study the effect of potential parameters, such as input power and concentration of magnetic susceptible material, on the selective heating in the microwave magnetic heating method. Microwave electric heating is commonly used in chemical synthesis, however, as the most of the materials in a reaction can interact with the microwave and absorb the microwave electric energy, the energy cannot travel into the centre of the bulk before dissipated (Galan et al., 2017; Amini et al., 2021). This very small penetration depth of microwaves poses design challenges in the scale up of microwave processes (Zhang et al., 2017; Morte et al., 2019). On the other hand, in the microwave magnetic heating, most of the organic materials do not possess any magnetic susceptibility, and will not compete in absorbing the magnetic field energy. In fact, most of the magnetic field energy are potentially consumed by the magnetic susceptible catalyst, and because of their low concentration compared to the bulk material (normally less than 5% wt in a reaction), the magnetic field energy has greater potentials to penetrate deeper into the bulk compared to the microwave electric heating. Therefore, this allows the process to bypass the penetration depth obstacle while maintaining the bespoken microwave heating effect even in a scaled-up process.

This paper describes the first experimental studies of applying a magnetic susceptible catalyst and the microwave magnetic heating for the polymerization of the ɛ-caprolactone. The characteristics, kinetics, and mechanism of the polymerization initiated by FeCl_3_ and Benzyl alcohol (BzOH) using microwave magnetic heating were reported and compared to identical reactions that were conducted with the conventional heating and the microwave electric heating methods. As PCL is commonly used for fabrication of biomaterials, the cytocompatibility of the produced polymer was investigated for future biological applications.

## 2 Experimental

### 2.1 Materials

FeCl_3_ (97% purity) was bought from Sigma Aldrich. The sample was dried in an oven at 70°C for 1 day before moving it into a desiccator for storage at room temperature. The ɛ-caprolactone monomers (97% purity) and Benzyl alcohol (98% purity) were bought from Sigma Aldrich without further purification. Fetal bovine serum (FBS) was bought from Zhejiang Tianhang Biotechnology Co., Ltd. Phosphate buffered saline was bought from Cytiva. Anhydrous ethanol (≥99.7%) was purchased from Shanghai Titan Scientific Co., Ltd.

### 2.2 Reactor geometries

In conventional heating (CH) ROP reactions, a standard oil bath was used where oil temperature was controlled by a thermocouple in the oil bath. The temperature was also cross-referenced to an internal bulk temperature measurement using an OF probe. A single-mode Sairem MiniFlow 200SS operating at 2.45 GHz was used as the microwave generator for both microwave electric heating (EH) and microwave magnetic heating (HH) experiments. All EH ROP reactions were conducted using the MiniFlow with a TE cavity equipped with an optic fibre (OF) probe thermometer. The OF probe was inserted directly into the reaction mixture for accurate and immediate temperature feedback. In HH ROP reactions, a MiniFlow with a TM cavity was used. An OF probe was again used for temperature detection. The procedure and validation of the heating samples at electric and magnetic dominant locations are described in the Appendix.

### 2.3 Heating experiment procedures

In heating experiments, 18 mg of FeCl_3_ was weighted and dissolved in 10 mL of the ɛ-caprolactone (CL) to make a solution of monomer to catalyst molar ratio ([M]:[C]) of 800:1. The solution was transferred into a reaction vessel for degasification. A quartz tube (diameter of 3 mm) with an open at the top was inserted into the rubber stopper on the sealed reaction vessel for the insertion of an OF thermometer, the sample was then sent for degasification. After degasification, the sample was sent to a TE cavity or a TM cavity for the EH or HH experiment.

### 2.4 ROP reaction procedure

In typical conventional heating (CH) ROP reaction, the monomer, catalyst, and initiator were weighted for specific [monomer]:[catalyst];[initiator] ([M]:[C]:[I]) ratio (i.e., 36 mg of FeCl_3_, 0.12 mL of BzOH, and 10 mL CL were weighted for [M]:[C]:[I] ratio of 400:1:5). These reactants were then transferred into separate reaction vessels, which was sealed with a rubber stopper, for degasification with argon. After 10 min of degasification, the monomer and initiator were transferred into a reaction vessel that contained the catalyst. The vessel was then immersed in an oil bath, which was preheated to the set temperature, to start the reaction.

During the kinetic study, 0.2 mL of the sample was sampled using a syringe. The obtained sample was transferred into a glass sample container and stored in the fridge at −20°C.

In the MWH reaction, identical preparation procedures as described in CH were conducted, but instead of sending to an oil bath, a TE cavity and a TM cavity were used for the EH and the HH, respectively. The internal bulk temperature was continuously monitored using an OF thermometer inserted directly into the reaction mixture *via* the quartz tube on the stopper. In these MWH experiments, the temperature measurements from OF probe were used to control the power input required to keep the bulk temperature constant at the target set point.

### 2.5 Analytical characterisation procedures

#### 2.5.1 Gel permeation chromatography (GPC)

GPC characterization experiments were performed on a Polymer Labs GPC-120 instrument at 35°C equipped with a PLgel 5 μm Guard column and two PLgel 5 μ MIXED-E columns in series coupled with a refractive index detector using HPLC grade THF as the mobile phase at a flow of 1.0 cm^3^ min^-1^. The GPC was calibrated with polystyrene narrow polydispersity index (Ð) standards close to 1.00. All GPC equipment and standards were supplied by Polymer Laboratories (Varian). GPC data was analysed using the Cirrus GPC offline software package.

#### 2.5.2 Nuclear magnetic resonance (NMR)

^1^H NMR spectra were recorded at 25°C using a Bruker DPX-300 spectrometer (300 MHz). Chemical shifts were recorded in δ_H_ (ppm). Samples are prepared as solutions in CDCl_3_. The monomer conversion was determined by comparing the integral of methylene proton resonance adjacent to the oxygen of the carbonyl group for the monomer (-CH2OCO-, δ = 4.24 ppm) and polymer (-CH2OCO-, δ = 4.07 ppm). An end-group analysis can also be done to identify the degree of polymerization (DP). It is done by comparing the integral of methylene proton resonance adjacent to the carbonyl group (H_x_, δ = 4.1 ppm) to that of methylene proton of benzyl alcohol (H_x_, δ = 5.1 ppm).

### 2.6 Cytotoxicity studies

The cytotoxicity of all the samples was determined by CCK-8 assay. The polymer sample produced with [M]:[C] ratio of 400:1 and 800:1 were first soaked with 75% ethanol solution for 2 h, then UV sterilized for 12 h 6 g of the sterilized sample was transferred into 6 mL of the culture media containing 15% fetal bovine serum (FBS) and soaked for 24 h to get leached out media. The soaked sample was centrifuged at 1000 rpm for 5 min and the supernatant was used for cell cytotoxicity studies.

A 5×10^3^ cells/well was seeded for 24 h in a 96-well plate and incubated at 37°C with 5% CO_2_. The media was then replaced with the prepared sample supernatant (100 µL). The plate was incubated at 37°C for 1 day, 3 days, and 5 days, accordingly in the CO_2_ incubator.

The media were disposed of at the end of the incubation and washed with PBS solution 3 times before adding serum-free medium containing 10% of CCK-8 (100 µL). The samples were then incubated in the CO_2_ incubator for 1 h. Absorbance was recorded using a Tecan Spark microplate reader at 450 nm.

## 3 Results and discussion

### 3.1 Heating experiments

**FIGURE 1:**
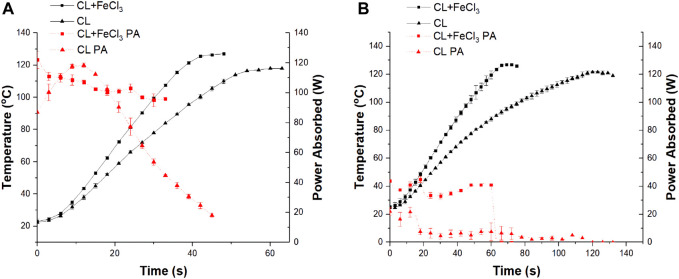
Example of typical temperature profile (black solid line) and power profile (red dot line) of CL and CL + FeCl_3_ mixture (n = 5) using **(A)** EH and **(B)** HH heating methods at 150 W input power. PA, power absorbed.

In our previous study, we identified that the presence of the catalyst could have a significant effect on the heating of the bulk (Wang et al., 2017). Therefore, a series of heating experiments were conducted first to identify if the chosen catalyst is susceptible to microwave electromagnetic heating (EMH) and can be reflected by the increment of the solvent bulk temperature. Two EMH heating methods were used: a) microwave E-field heating (EH), where the E-field is at a dominant position, and b) microwave H-field heating (HH), where the H-field is at a dominant position. However, it should be noticed that although it is heated at an E-field or an H-field dominant position, there is still a presence of the other field heating the sample. For example, a weak E-field is still present when conducting an H-field heating experiment, and *vice versa*. No initiators were added to the system ensuring no polymerization occurs during the heating experiment. The temperature profile was monitored with time by the inserted optic fibre (OF) temperature sensor in the sample. The OF temperature sensor was reported to be able to achieve a direct measurement of the reaction medium bulk temperature by previous researchers (Robinson et al., 2010a; Robinson et al., 2010b; Adlington et al., 2014; Nguyen et al., 2014). The maximum input power was set to 150 W for all experiments unless mentioned otherwise. The temperature profiles and power profiles of the heating experiments were recorded by the MiniFlow and were shown in Figure 1 for both the EH and HH heating methods.

Figure 1 shows the temperature profiles and absorbed power variation with time during the EMH experiments for all samples. The power absorption was calculated as the difference between the incident and the reflected power during the experiment. It must be noted that impedance matching was only carried out during the onset of the heating trial and was not maintained continuously throughout the experiment. Therefore, the power absorption data may also be influenced by any differences in the reflected power due to impedance “mismatch” that potentially takes place during the duration of the experiment as the samples are heated up. As the result, a constant decreasing trend in the absorbed power profile was observed for all samples.

Figure 1A demonstrated the heating profile of the neat CL monomer on its own when no FeCl_3_ or initiator was present, and the identical monomer with FeCl_3_. The neat monomer was able to be heated efficiently with the E-field heating. However, when FeCl_3_ was present in the monomer, the heating of the bulk was enhanced, i.e., a temperature difference of up to 20°C was obtained compared to the neat monomer at the same time mark. This temperature difference along with the additional 19 W absorbed by the FeCl_3_ sample suggested that this additional power should be absorbed by FeCl_3_ and distributed within the bulk to result in such temperature differences.

Meanwhile, reviewing the heating profile of the same samples in the H-field heating as shown in Figure 1B an even greater temperature difference (up to 33°C) was observed with a similar amount of additional power absorbed (around 20 W) by a the FeCl_3_ sample in comparison to the EH heating experiment. This greater temperature difference was detected by the HH heating method, indicating a significantly stronger magnetic selective heating from the presence of the catalyst.

It should be reminded that the catalyst used in these heating experiments were in the molar ratio of 1:800 to the bulk media. With this little amount of the catalyst used, the catalyst must underwent excessive heating to contribute such observed temperature differences.

**FIGURE 2:**
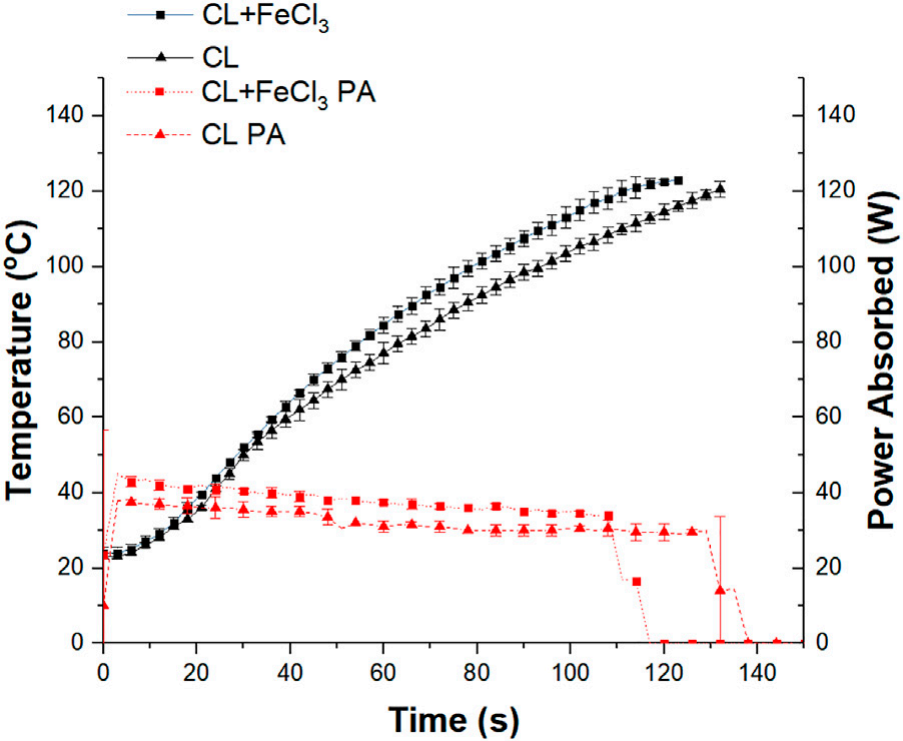
Temperature profile (black solid line) and power profile (red dot line) of CL and CL + FeCl_3_ mixture (n = 5) using EH at 50 W input power.

However, it should be noted that the MiniFlow can only detect the E-field energy that is being put into and reflected, ignoring the presence of the H-field energy. As the sample was located at the H-field dominant position where only a small amount of energy from the weak E-field was absorbed by the sample, and thus the reading of the power profiles was found to be lower compared to the EH heating experiment. But it should be reminded that this weak E-field heating together with the strong H-field heating contributes to the heating of the bulk in these HH experiments. To identify if the observed temperature difference purely originated from the weak E-field heating, another series of low-power EH experiments were conducted, and the temperature and power profiles were shown in Figure 2.

In Figure 2, the CL sample took approximately 125 s to reach 120°C which was similar to the time required for CL in the H-field heating experiment shown in Figure 1B (120 s–120°C), this suggested that both samples should be experiencing a similar level of the E-field heating. Additionally, the power absorbed by the CL + FeCl_3_ sample (≈40 W) is similar to that in the HH experiment at the beginning of the experiment. However, this time, the CL + FeCl_3_ sample took around 120 s to reach 120°C rather than 60 s for the HH experiment. This obvious difference between the two heating profiles confirms that the FeCl_3_ should be experiencing significant H-field heating in the HH experiment and resulted in such heating enhancement of the bulk.

On the other hand, from this weak E-field heating experiment, it was also realized that the temperature differences observed in the EH experiment from Figure 1A were not reproduced in Figure 2. This suggested that such observed temperature difference in the EH experiment was only obvious at high input power levels.

From these heating experiments, it was noticed that the heating of the neat CL in the HH required an additional 60 s to reach the set temperature compared to the EH heating method, this was because the CL sample was only heated by the weak E-field present in the HH. Conversely, for the CL + FeCl_3_ sample, it only required an additional 15 s to reach the set temperature compared to the EH heating method. Such a significant reduction in heating time (from 120 s to 60 s) in the heating of CL + FeCl_3_ sample suggested that the magnetic susceptible FeCl_3_ was significantly heated from the H-field so that it was able to achieve a similar heating performance compared to the EH. In the H-field heating, rather than a significant portion of the heating from the E-field, it should be that the catalyst was being selectively heated by the H-field and only a relatively minor part, in this case, was related to the weak E-field heating to the bulk.

From the heating experiments, we have demonstrated that the catalyst must experienced significant selective heating effects to contribute such different bulk heating performance, as the catalyst concentration used was only 0.125% molar ratio.

### 3.2 ROP reactions with various heating methods

A key aim of the present study was to define the effect that the presence of the selective heating effect on the catalyst has upon the performance of its catalytic activity in polymerization reactions. Thus, bulk ring-opening polymerization (ROP) of CL was conducted next using CH, EH, and HH heating methods. BzOH was chosen as the initiator because it is the most common alcohol used as the initiator in ROP reactions. Additionally, Hege and Schiller found that ROP using FeCl_3_ performed best with BzOH as the initiator (Hege and Schiller, 2014). Therefore, BzOH was selected to be the initiator for all ROP reactions.

The influences of reaction conditions, including temperature, monomer to catalyst molar ratio ([M] [C] ratio), and input power was investigated. A wide range of temperatures from 50 to 150°C was selected, the latter of which is a typical operation temperature for ROP polymerization. In this study, three [M]:[C] ratios were used 400:1, 800:1, and 1600:1. and six different input power were studied, which were 25, 50, 75, 100, 125, and 150 W.

#### 3.2.1 Effect of temperature

The effect of temperature on the bulk polymerization of CL initiated by FeCl_3_ and BzOH was first investigated as shown in Table 1. At 50°C, a 60% conversion was achieved after 25 min by using the CH (Table 1 entry 1). The temperature was then increased to 75°C, and the polymerization reactions were able to achieve 97% conversion within 25 min (Table 1 entry 4). However, as the temperature was further increased to 100, 125, and 150°C, the polydispersity (Ð) started to get broaden, and the conversion dropped to 84%, 64%, and 62%, respectively at 25 min mark (Table 1 entry 7, 10, 13). This was due to the undesired transesterification side reactions taking place at elevated temperatures or at long reaction times leading to the formation of cyclic polymer or “back-biting” (Gong et al., 2021). As a result, the Ð value increased as the temperature increased as shown in Table 1 as well as from GPC trace shown in the support document Supplementary Figure S6).

**TABLE 1:** Average results of ROP of CL with FeCl_3_ and BzOH at various temperature at [M]:[C]:[I] ratio of 400:1:5.

Entry	Temperature (°C)	time (min)	Heating method	Mn^a^ (g mol^−1^)	Mp^a^ (g mol^−1^)	Ð^a^	Conversion (%)
1	50	25	CH	3200	5000	1.42	59.2
2	50	25	EH	3500	5400	1.27	88.5
3	50	25	HH	4700	6600	1.37	91.5
4	75	25	CH	3900	6300	1.43	97.0
5	75	25	EH	4500	6200	1.33	99.9
6	75	25	HH	5000	8000	1.39	99.9
7	100	25	CH	3700	6700	1.31	84.3
8	100	25	EH	4200	8300	1.68	99.9
9	100	25	HH	4700	9700	1.55	96.3
10	125	25	CH	2500	5300	1.66	64.2
11	125	25	EH	3100	7100	1.97	76.6
12	125	25	HH	4300	9400	1.66	83.7
13	150	25	CH	1900	4900	1.81	62.1
14	150	25	EH	2700	6200	1.87	73.3
15	150	25	HH	3100	7900	1.83	81.7

^a^ Determined by GPC, measured in THF, at 35°C.

^b^ Determined by ^1^H-NMR.

These results from the CH experiment were then compared to those conducted with electromagnetic heating (EMH) including the EH and HH heating methods. It was observed that changing from the CH to the EMH, the conversion of the ROP at 50°C for 25 min was increased from 59% in the CH to 88% and 91% in the EH and HH heating methods, respectively (Table 1 Entry 1, 2 and 3). However, the EMH experiments followed the same trend as CH experiments that the product reached peak Mwt at 100°C, and started to show a reduction in the conversion and broadening in Ð at elevated temperatures.

Directly comparing Mwt results from the CH and the EMH, the EMH results showed improvements in the Mwt of produced polymer, in terms of M_n_ and M_p_. e.g., at 150°C, the M_n_ value for the EH and the HH at 25 min were 2,700 and 3100 g mol^-1^ which was 35% and 55% higher than that in the CH. Improvement in Mwt was found to be more significant in HH than EH (typically ∼15%–33% greater at each point compared to the EH results). This was hypothesized to be due to the difference in the heating mechanism as found in heating experiments, where both the monomer and the catalyst are susceptible to strong E-field heating, but only the catalyst is susceptible to strong H-field heating. Such differences in the heating could result in selective heating of the species that potentially affect the initiation and/or the propagation step of the polymerization. The heat concentrated around the monomer and/or the catalyst due to the selective heating could potentially promote the polymerization on the site and enhance the initiation and/or propagation.

**FIGURE 3:**
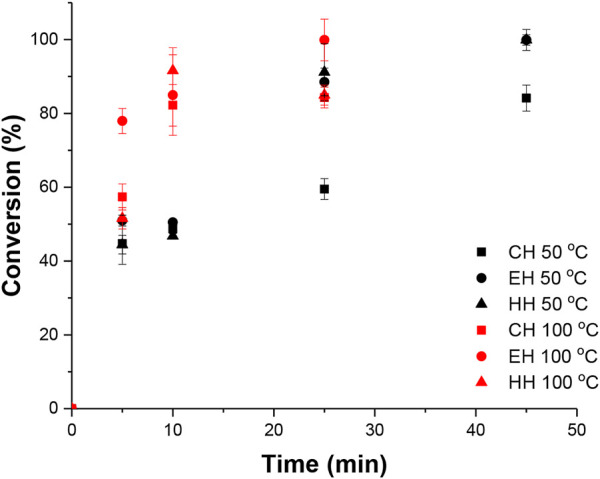
Comparison of conversion versus time plots for ROP of CL in CH (n = 3) using FeCl_3_ between 50°C and 100°C.

To further elaborate on the effects of temperature on the polymerization, a series of kinetic studies were then conducted with three heating methods, detailed kinetic study plots are shown in the supporting documents Supplementary Figures S7–S9. Figure 3 demonstrates a comparison of kinetic studies between 50 and 100°C with the CH, the EH, and the HH. The blue markers are for reactions at 50°C and the red markers are for that at 100°C.

Inspecting Figure 3, when using 50°C as the reaction temperature, the initial reaction conversion was similar among the three heating methods up to 25 min mark. Whereas at 100°C, significant conversion differences were observed from 5 min mark (above 80% conversion for EMH heating methods and only around 55% for the CH heating method). This could potentially be a combination of higher reaction temperature and excessive exposure to microwave energy at the beginning of the heating step. A large amount of energy (up to 150 W) was being put into the system at the beginning of the reaction, and the time that the sample was exposed to such an amount of power before reaching the set temperature was significantly different for different reaction temperatures. The effects of microwave energy were investigated and will be discussed in later sections.

Based on the temperature study, we have identified that the raised reaction temperature can significantly accelerate the polymer propagation. In fact, in combination with magnetic selective heating effects, the catalyst reaction site would be at an elevated temperature to further accelerate the propagation rate, and thus a higher M_p_ results were obtained for HH experiments which are 17%–33% greater than the EH experiment at each reaction temperature.

#### 3.2.2 Activation energy calculation

The previous empirical results suggested that the application of EMH heating methods promotes the reaction significantly, therefore, to identify if this purely originated from thermal effects, the activation energy required for the polymerization was studied next.

To do this, the rate of propagation (K_app_) for each heating method at various temperature were calculated based on the kinetic plot of ln (M_0_/M) *versus* reaction time conducted various temperatures. Kinetic plots for the CH, EH, and HH experiments can be found in the supporting document Supplementary Figures S10–S12, respectively. From the plots, the K_app_ value can be calculated by identifying the slope of the plot for first-order reactions. The summary of the K_app_ values is shown in Table 2.

**TABLE 2:** Summary of the rate constant (Kapp) at different temperatures using CH, EH, and HH. Condition [CL]:[FeCl_3_] = 400:1.

Temperature (°C)	CH	EH	HH
	kapp (min^−1^)	kapp (min^−1^)	kapp (min^−1^)
50	0.035	0.069	0.059
75	0.130	0.156	0.154
100	0.173	0.332	0.279

Analysis of the kinetic plots, a straight-line relationship holds for ln (M_0_)/M as a function of reaction time at 50–100°C. This linear relationship demonstrated that polymerization is a controlled first-order reaction. However, this relationship deviated from the trend line at 125 and 150°C. This was due to the competition in side reactions causing the reaction to losing its control. Therefore, only the controlled first-order reactions were selected to calculate the polymerization rate constant (K_app_), based on the gradient of the kinetic plots from the ROP reactions and is summarised in Table 2.

Inspecting the data in Table 2 led to the conclusion that the K_app_ value of all the EH and the HH was at least 1.4 and 1.1 times higher than that from the CH experiment at the same bulk temperature. This again shows that the presence of selective heating in the EMH promotes the polymerization rate, as the EH heats both the monomer and the catalyst (reaction site) directly, while the HH primarily heats the catalyst. Both heating methods resulted in concentrated local heating above the measured bulk temperature which facilitated the reaction rate.

**FIGURE 4:**
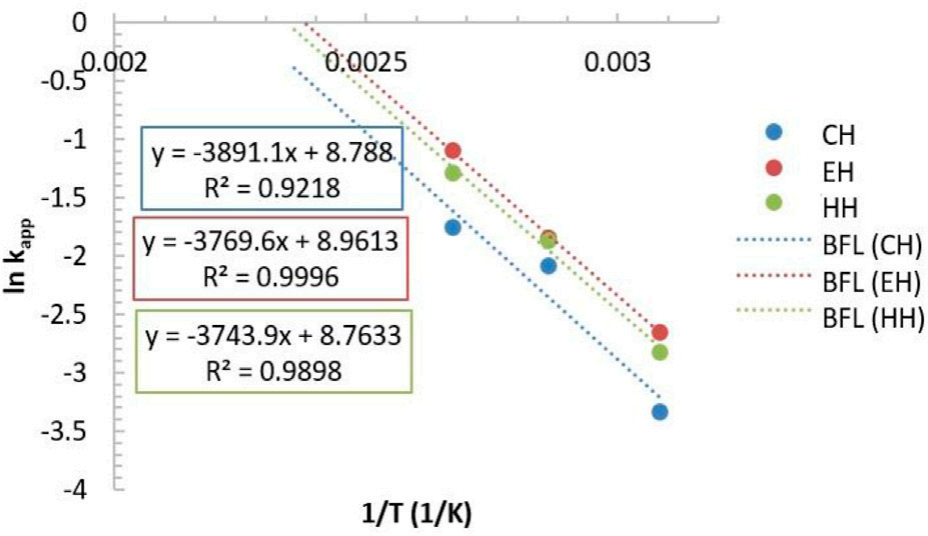
The relationship between lnKapp versus the reciprocal of absolute temperature. Condition [CL]: [FeCl_3_] = 400:1.

The activation energy of the polymerization was then established based on these temperatures by plotting ln (K_app_) against 1/T for all heating methods. The Arrhenius plot is shown in Figure 4.

The Arrhenius plot was noted to have similar gradients among the three heating methods. From the plot, it was able to calculate the activation energy (E_a_) using the Arrhenius equation. It was found that there were no significant differences in the E_a_ between all heating methods. Indeed, the E_a_ calculated for the CH, the EH, and the HH was 32.5, 31.3, and 31.1 kJ/mol, respectively. Such results suggested that the mechanism of the reaction was not altered by different heating methods. The observed differences in the K_app_ when the EMH was applied purely originated from thermal effects. The EMH heating methods potentially created heat concentration around/at the catalyst *via* selective heating, and accelerated the polymerisation of the monomer.

#### 3.2.3 Effects of microwave electromagnetic energy

As previously mentioned, in a typical EMH experiment, the MiniFlow starts with putting maximum microwave EM energy (150 W) to the bulk to elevate the temperature to the desired point, once reaching and holding at the temperature, only minimized amount of energy was applied. In addition, a higher power would be required to hold at a higher temperature due to increased heat loss to the surrounding, an example of a typical temperature and power profile for EMH reaction can be found in the supporting document Supplementary Figure S13. Therefore, for reactions at 100°C, the bulk spent a long time (around 50 s) under high-intensity EM energy (150 W) compared to that at 50°C (around 20 s). In addition, the power required to maintain at 50°C and 100°C were different (around 10 W and 30 W for 50°C and 100°C, respectively). These empirical observations be the potential reason for the observed conversion differences at the beginning of the reaction at different temperatures.

To further understand this, the effects of different EM energy/power were then studied. Short reactions at different input powers with both the EH and the HH heating methods were studied. These reactions were conducted for 180 s at 100°C for all samples. The characteristics and yield data of polymer products for the set of reactions were contained in Tables 3, 4 for the EH and HH experiment, respectively.

**TABLE 3:** Average results of ROP of CL with FeCl_3_ and BzOH using various input power at [M]:[C] ratio of 400:1 for 3 min with EH.

Input power (W)	Mn^a^ (g mol^−1^)	Mp^a^ (g mol^−1^)	Ð^a^	Conversion (%)
25W	2100	2630	1.19	54.33
50W	2300	2900	1.21	65.63
75W	1620	2750	1.5	64.15
100W	2430	3040	1.232	71.67
125W	2460	3110	1.231	76.41
150W	2260	3080	1.31	79.67

^a^ Determined by GPC, measured in THF, at 35°C.

^b^ Determined by.^1^H-NMR.

**TABLE 4:** Average results of ROP of CL with FeCl_3_ and BzOH using various input power at [M]:[C] ratio of 400:1 for 3 min with HH.

Input power (W)	Mn^a^ (g mol^−1^)	Mp^a^ (g mol^−1^)	Ð^a^	Conversion (%)
25W	2090	2070	1.41	50.73
50W	2210	3400	1.39	55.15
75W	2580	4360	1.38	67.32
100W	2700	4000	1.31	82.30
125W	2700	4060	1.53	82.72
150W	3020	3870	1.37	83.22

^a^ Determined by GPC, measured in THF, at 35°C.

^b^ Determined by.^1^H-NMR.

**FIGURE 5:**
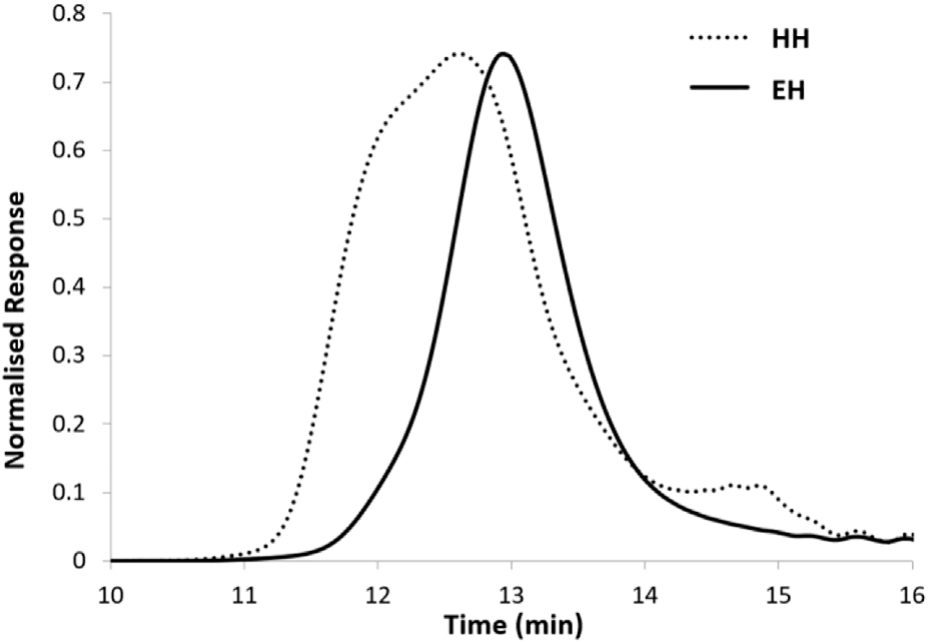
Comparison of the GPC trace of the product polymer synthesized with identical reaction time under E-field (solid line) and H-field (dot line) heating at a power of 150 W. Condition [CL]:[FeCl_3_] = 400:1.

Comparing the results from Table 3 to Table 4, at low input power (at 25 and 50 W), both EH and HH heating results were very similar in both yield and product polymer Mwt characteristics. However, by putting in more power, the differences in the conversion and Mwt became more significant between the two heating methods at identical conditions, i.e., up to 60% differences in M_n_ and 15% in conversion. Further inspecting the GPC trace from Figure 5, PCL produced from the HH at 150 W input power showed a signal trace from 10.9 min, whereas the GPC trace for the EH started from around 11.5 min. This difference in signal detection suggested that the Mwt of polymer produced from the HH was significantly higher than that obtained with the EH. These observations exhibited an identical trend as previous ROP studies showed in Table 1 where higher PCL Mwt were obtained with the HH than that with the EH, and a broadened GPC peak was found for the HH product than the EH product.

These scenarios showed that the differentiation between HH and EH in terms of Mwt and conversion was more significant at high input power. In the EH heating method, both the monomer and the catalyst compete in absorbing the E-field energy, therefore, the selective heating effect on the catalyst is not maximized. However, in the HH heating scenario, only the magnetic susceptible catalyst can absorb the H-field energy from the alternating EM field, this allows the catalyst to experience a stronger concentrated local heating compared to the EH heating method. This stronger magnetic selective heating effect provides a rapid increase in reaction temperature and achieves a higher local temperature than the bulk. As previously discussed, due to the selective heating of the catalyst in the HH experiment, the reaction site temperature could be significantly higher, and therefore caused the acceleration in the ROP and the side reactions which resulted in a higher product Mwt and a broadened peak distribution as we identified in the temperature study.

#### 3.2.4 Effects of catalyst concentration

Previous studies have shown that if the species that is being selectively heated is presented in too small a quantity, the predominant heating effects from EMH would be diminished (Adlington et al., 2014). Therefore, ROP reactions with reduced catalyst load were also studied at 100°C, to investigate if the observed enhancement in the HH heating method would diminish at lower catalyst concentration. The obtained results are shown in Table 5, and the detailed results for the ROP at different catalyst loads at various temperatures were summarised in supporting documents Supplementary Tables S2, S3.

**TABLE 5:** Average results of ROP of CL with FeCl_3_ and BzOH at various [M]:[C] ratios at 100°C.

Entry	[M]:[C]	Time (min)	Heating method	M_n_ ^a^ (gmol^−1^)	M_p_ ^a^ (gmol^−1^)	Ð^a^	Conversion^b^ (%)
1	400:1	25	CH	3700	6700	1.31	84.3
2	400:1	25	EH	4200	8300	1.68	99.9
3	400:1	25	HH	4700	9700	1.55	96.3
4	800:1	45	CH	5000	7900	1.56	63.6
5	800:1	45	EH	5700	9800	1.46	76.4
6	800:1	45	HH	6300	11000	1.44	79.1
7	1600:1	120	CH	6700	8900	1.23	51.7
8	1600:1	120	EH	7500	10100	1.20	71.1
9	1600:1	120	HH	7500	9700	1.24	67.9

^a^ Determined by GPC, measured in THF, at 35 C.

^b^ Determined by.^1^H-NMR.

Inspecting Table 5, reducing the catalyst concentration significantly slow down the reaction because fewer reaction sites are available for the polymerisation, and the reaction time was extended from 25 to 120 min. In the meantime, more monomers were able to attach to a polymer chain, and thus, higher Mwt results were able to be achieved at identical conversion.

It was notice that Mwt results for the HH heating method were superior than the EH results at [M]:[C] ratios of 400:1 and 800:1, but such difference was diminished as the catalyst load was reduced to [M]:[C] ratio of 1600:1. For example, at [M]:[C] ratio of 400:1 and 800:1, the M_p_ results for the HH experiments were 17% and 12% higher than the EH experiments, respectively; but at [M]:[C] ratio of 1600:1, the M_p_ result for EH experiment is 4% greater than the HH experiment. This suggested that the reduction in the catalyst was more significant in the HH than the EH, as the FeCl_3_ is the main species that is susceptible to the H-field heating.

If a specie that undergoes the magnetic selective heating is too small in quantity, the predominant heating method remains as EH due to the presence of the electric field. As a result, comparable product Mwt and conversion between the EH and the HH were obtained. This observation agrees with our previous study; (Wang et al., 2017); however, it should be noted that although no superior Mwt and conversion were obtained from the HH heating method, comparable results were still obtainable compared to the EH heating method. Considering that as both monomer and catalyst absorb the E-field energy, less energy can travel to the centre of the bulk and result in a small penetration depth in the EH heating method. On the other hand, only the catalyst which is in a small amount is absorbing the H-field energy, the H-field energy would be able to travel further into the bulk, therefore, providing more opportunities in scaling up the process and potentially overcome the design challenges in the EH heating methods related to the penetration depth.

### 3.3 Cytotoxicity studies

**FIGURE 6:**
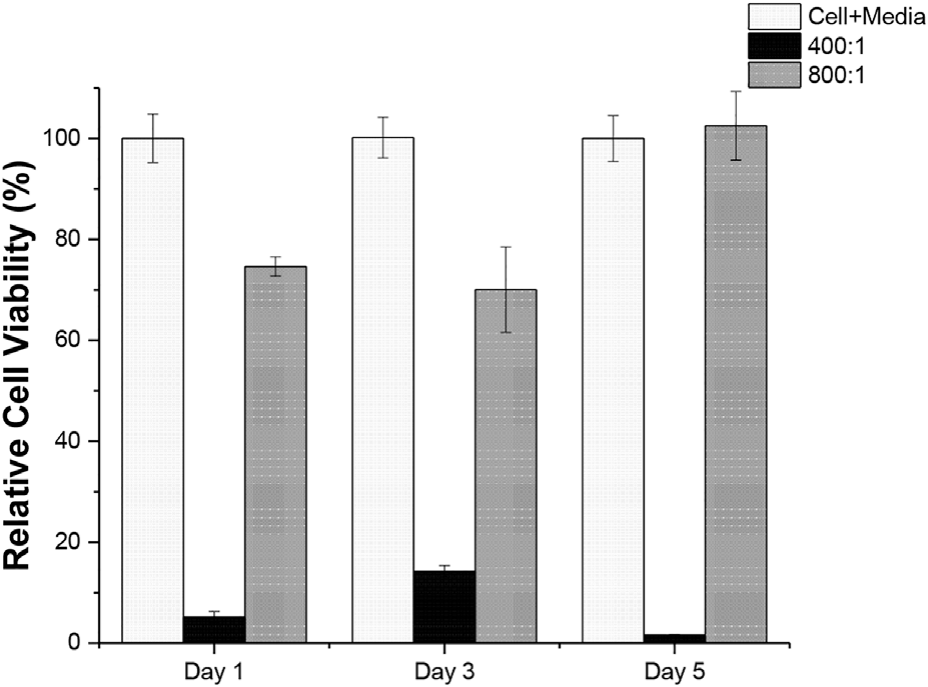
Percent cell viability of Panc02 cells at day 1, 3, and 5 (n = 5) for Cell + Media sample and PCL polymer produced with [M]:[C] ratio of 400:1 and 800:1. Values are expressed as relative % viability; mean ± SD.

CCK-8 cell viability assay was performed to assess the polymer cytotoxicity and the results were summarised in Figure 6. As shown in the Figure, it is very obvious that a distinct cell toxicity between the polymer produced with [M]:[C] ratio of 400:1 and 800:1. For PCL produced with [M]:[C] ratio of 800:1, the cell viability maintains above 70% throughout the testing period, suggesting that the polymer remains non-toxic to cells. On the other hand, the polymer produced with [M]:[C] ratio of 400:1 has a detrimental effect to cell over the testing period. This was due to a higher concentration of the catalyst used in the synthesis.

Although FeCl_3_ was can be used in fabrication of biocompatible materials (Petrenko et al., 2011; Hege and Schiller, 2014), the cytotoxicity of the produced material still needs to be assessed. The result shows that the amount of FeCl_3_ needs to be controlled properly as a high FeCl_3_ load ([M]:[C] ratio of 400:1) condition is harmful to cells, but if controlled at a proper level (i.e., at [M]:[C] ratio of 800:1) the produced PCL is non-toxic to the cell and can be potentially used as cyto-compatible materials.

## 4 Conclusion

For the first time, microwave magnetic heating (HH), where the magnetic field (H-field) from an electromagnetic field is dominant, is applied to the bulk ROPs of a lactone monomer with a magnetic susceptible and biocompatible catalyst. Superior Mwt were found in the HH at a high catalyst load compared to EH and CH methods, indicating the efficiency of the magnetic susceptible catalyst was enhanced by the HH. However, the activation energy for all heating methods was similar, suggesting the polymerization mechanism was not affected by the heating method and the observed differences in the EMH methods purely originated from thermal effects. Reducing the catalyst load diminishes the Mwt differences between the EH and HH methods. This could potentially be due to the species that are susceptible to the H-field heating being present in smaller quantities. In such case, the selective H-field heating could not provide a superior concentrated local heating than the EH, but comparable results were still achievable in the HH at low catalyst load, suggesting that the HH could still be a potential alternative heating method to the EH. Cytocompatibility studies showed the produced PCL is harmless when the catalyst load is controlled at the proper level.

## Data Availability

The original contributions presented in the study are included in the article/Supplementary Material, further inquiries can be directed to the corresponding authors.
