# Supplementary material for: Ring opening polymerisation of ɛ-caprolactone with novel microwave magnetic heating and cyto-compatible catalyst
Source: Front Bioeng Biotechnol. 2023 Feb 13;11:1123477. doi: 10.3389/fbioe.2023.1123477 (PMC9968877; doi:10.3389/fbioe.2023.1123477)
Supplement: Supplementary file 1 [file DataSheet1.docx]

**Ring Opening Polymerisation of ɛ-Caprolactone with Novel Microwave Magnetic Heating and Cyto-compatible Catalyst**

*Kaiyang Wang^1^, Ming Ni^2*^, Adam A. Dundas^3^, Georgios Dimitrakis^4^, Derek J. Irvine^3*^*

^1^ Shanghai Engineering Technology Research Center for Pharmaceutical Intelligent Equipment, Shanghai Frontiers Science Center for Druggability of Cardiovascular Non-coding RNA, Institute for Frontier Medical Technology, Shanghai University of Engineering Science, Shanghai, 201620, P. R. China

^2^ Department of Orthopaedics, Shanghai Key Laboratory for Prevention and Treatment of Bone and Joint Diseases, Shanghai Institute of Traumatology and Orthopaedics, Ruijin Hospital, Shanghai Jiao Tong University School of Medicine, Shanghai 200025, P. R. China

^3^ Centre for Additive Manufacturing, Faculty of Engineering, University of Nottingham, NG7 2RD, United Kingdom

^4^ George Green Institute for Electromagnetics Research, Faculty of Engineering, University of Nottingham, Nottingham, NG7 2RD, United Kingdom

*** Correspondence:**

Derek Irvine: [Derek.Irvine@nottingham.ac.uk](mailto:Derek.Irvine@nottingham.ac.uk)

Ming Ni: gendianqing@163.com

**Keywords: Microwave synthesis; Microwave selective heating; Magnetic susceptible catalyst; Biomaterial fabrication; PCL synthesis; Controlled polymerisation**

**Positioning in electric field dominant and magnetic field dominant position**


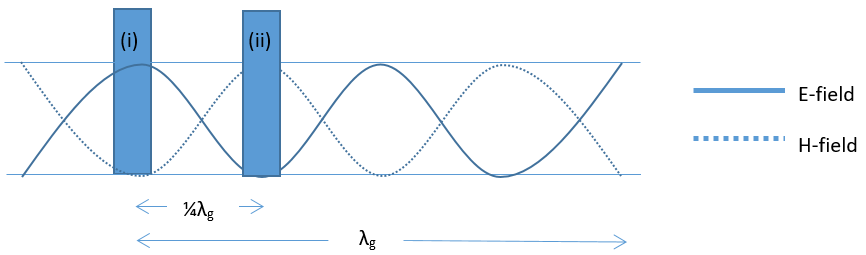


Figure S1 Details of the tube positioning in a single-mode microwave resonator: (i) maximal position of the E-field density and (ii) maximal position of the H-field density.

The sample positioning for heating experiments in a single mode transvers electric (TE) cavity is illustrated as showed in Figure S1. The maximal position of E-field density was obtained by tuning the short circuit until the lowest reflected power was obtained Figure S1 (i)). The maximal position of H-field was then obtained by shifting the maximal E-field position by quarter of the microwave wavelength (λ_g_) as showed in Figure S1. The MiniFlow used in this study is a standard WR-340 waveguide (4.3cm×8.6cm), which gives an electromagnetic field with a group wavelength (λg) of 174.28 mm at 2.45 GHz. [1] Therefore, the short circuit was shifted by 43.57 mm to allocate the sample at maximal H-field density (Figure S1 (ii)).

However, due to the tuning process in the TE cavity, two separate tuning processes were required for the magnetic heating experiments to achieve the magnetic field dominant position. This could potentially introduce experimental errors and inconsistent results were obtained. To avoid this, a single mode TM cavity was used for the magnetic heating experiments.

In the single mode TM cavity, the maximum level of the electric field was located in the centre of the cavity. However, as the sample was inserted to the centre of the cavity without tuning, due to its different dielectric property, the maximum electric field shifted and the magnetic component of the electromagnetic field was believed to become dominant. To justify if the sample was located at magnetic field dominant position, a simulation about field propagation in the TM cavity was done, and magnetic heating experiments comparing the heating between TE cavity at magnetic dominant position and TM cavity were also conducted.

The simulation was performed using CONCERTO, and was ran on a Lenovo Thinkpad with intel core i5-42000U 1.6 GHz processor and 8 GB RAM. The schematic of the geometry of the TM cavity was shown in Figure S2 with 3,000,000 cells for 30,000+5,000 iterations each. The input power used in the simulation was 100 W at 2.45 GHz. The metal part of the cavity was assumed to be perfect conductor, and the physical properties of ε-caprolactone used in the simulation were summarised in Table S1.

**Table S1 Physical properties of ε-caprolactone**

| ε’ | 27.48 @150 ^o^C |
| --- | --- |
| ε’’ | 0.153 @150 ^o^C |
| Heat capacity | 196.8 J/mol K @20 ^o^C |
| Density | 1.03 g/cm^3^ |


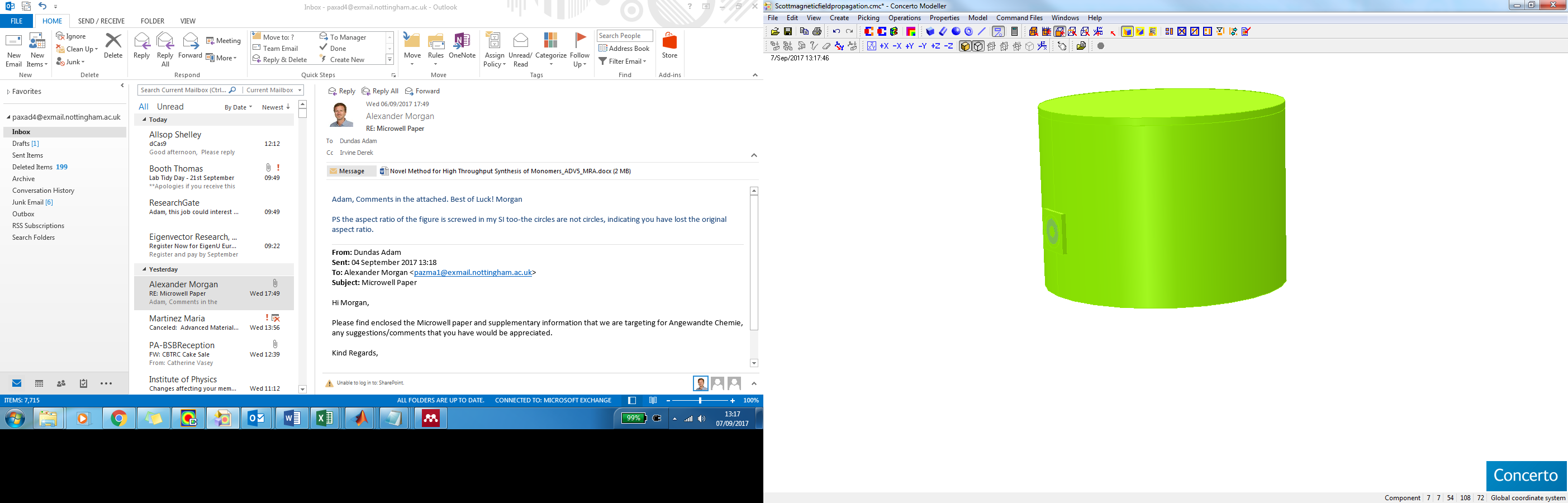

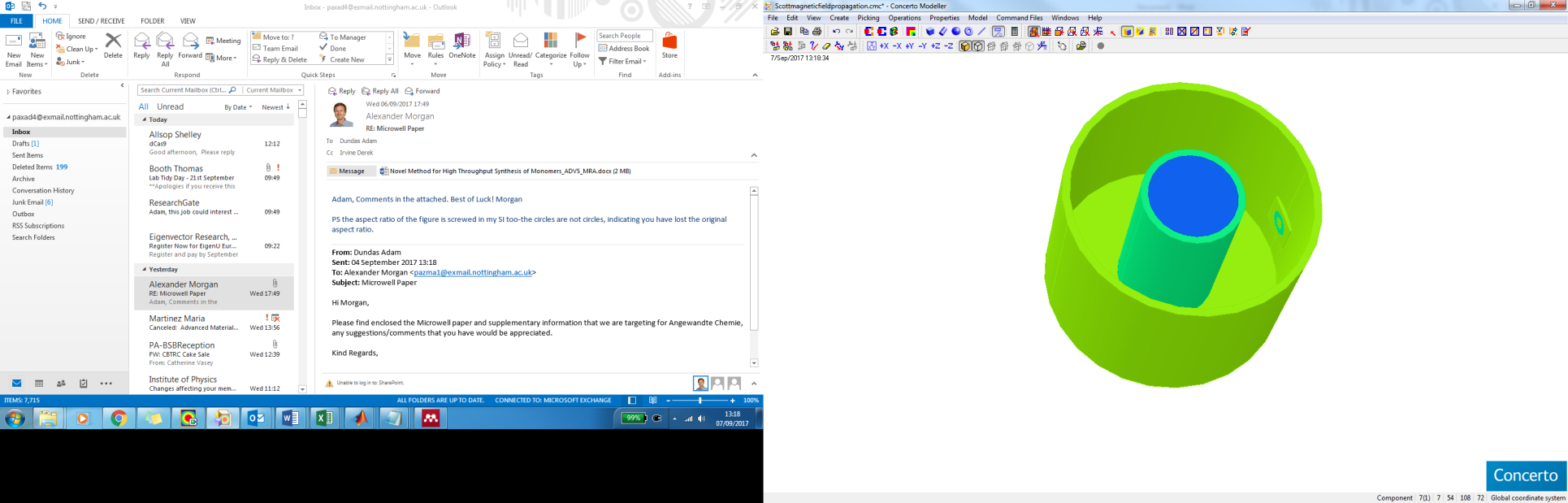


Figure S2 Schematic of the geometry of the TM cavity. Lime green shows metal, light blue shows PTFE and dark blue shows ε-caprolactone.

First, the simulation of the propagation of the E-field was performed as shown in Figure S3, whereas the simulation for the propagation of the H-field was shown in Figure S4.


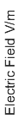

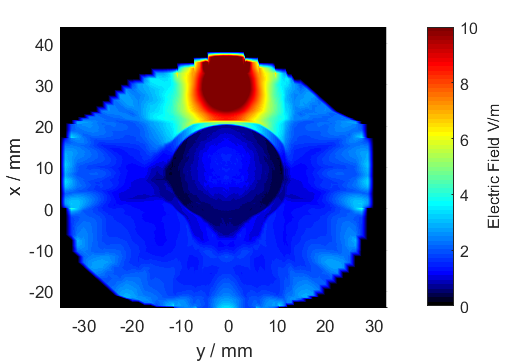

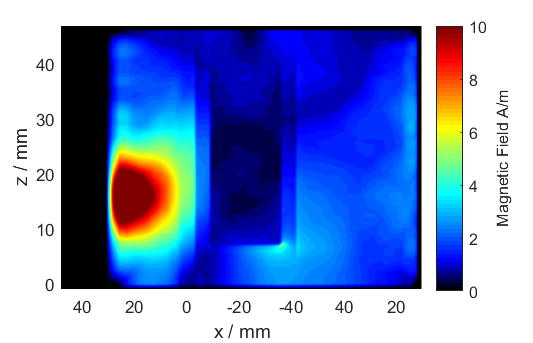


Figure S3 E-field propagation slice taken at XY = 25 mm and XZ = -10 mm, Left: a ‘birds-eye’ view of the geometry, right: a side view of the geometry.

From the figures above, it was obvious that most of the E-field propagated around the inlet of the energy source, and was not propagating through the sample at the centre as shown by the dark regions at the centre of the cavity. This simulation clearly demonstrated that there were no E-field propagated within the sample in the TM cavity.


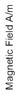

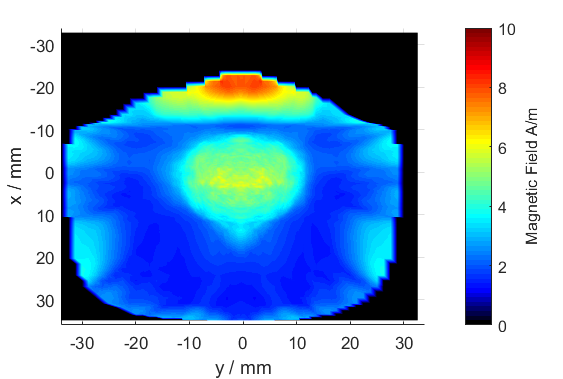

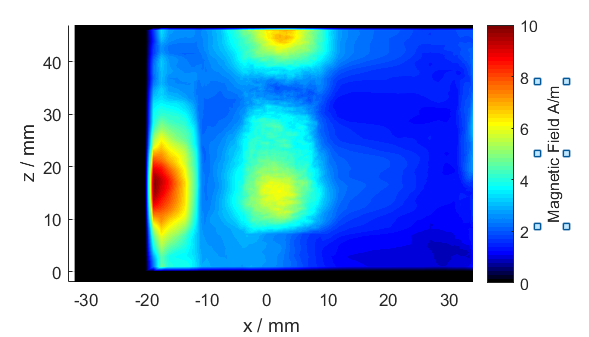


Figure S4 H-field propagation slice taken at XY = 25 mm and XZ = -10 mm, Left: a ‘birds-eye’ view of the geometry, right: a side view of the geometry.

Inspecting Figure S4, the simulation showed that the H-field propagated around the inlet of the energy source, as well as at the centre of the cavity where the sample located. And this suggested that a substantial amount of magnetic energy was presented within the sample when heating the sample in the TM cavity. Combining the simulation results of both E- and H-field, it showed that when performing the microwave heating in the TM cavity, the heating of the sample should primarily be magnetic heating.

A simulation of the power density within the cavity was also shown in Figure S5.


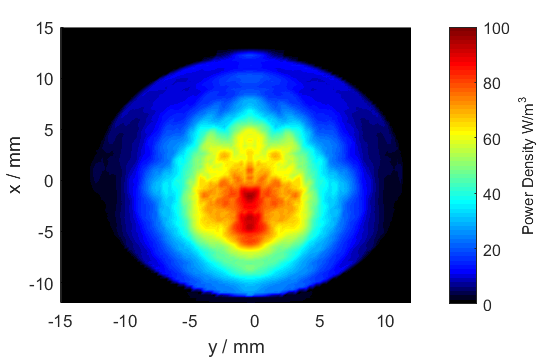

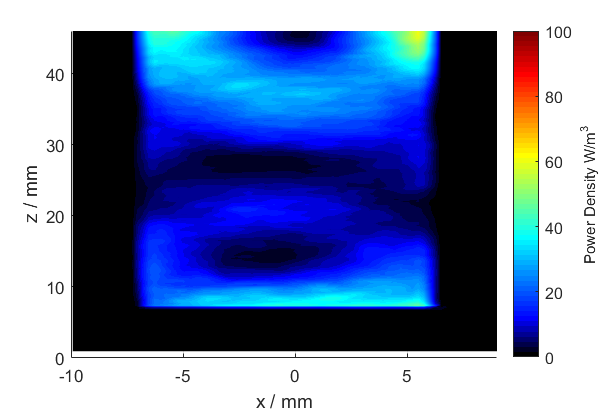


Figure S5 Power density plot for the TM cavity at XY=25 mm and XZ= -10 mm. Left: a ‘birds-eye’ view of the geometry, right: a side view of the geometry.

The power density figures above showed that the power density at the area where the sample located was at the highest, suggesting that the most of the energy was focused at the location where the sample was placed.

From the simulations, it was found that the heating of the sample in the TM cavity should mainly be the magnetic heating.

**Supporting Graphs for Results Section**


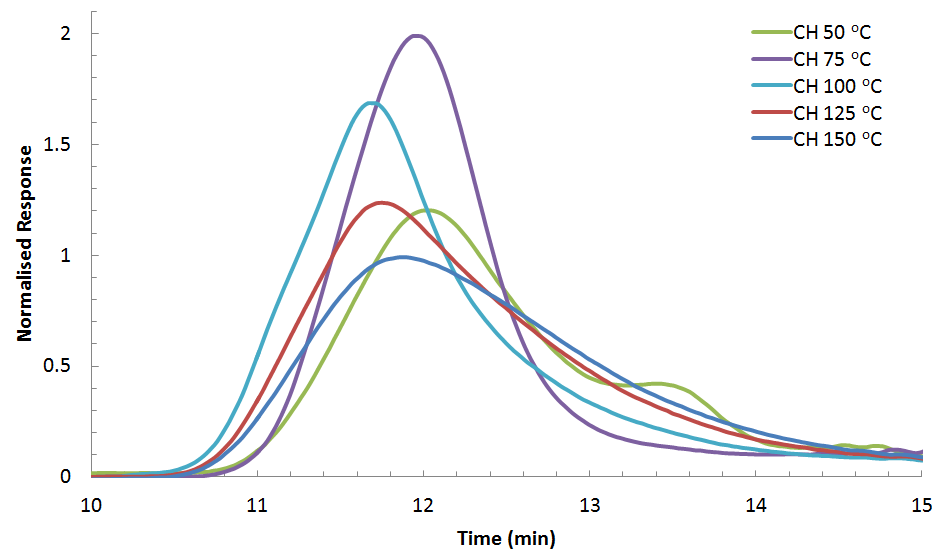


Figure S6 Comparison of the GPC traces of the product polymer synthesised using FeCl_3_ at different temperatures


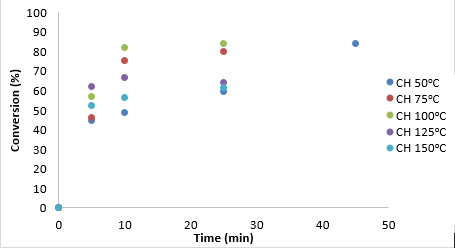


Figure S7 Conversion versus time plots for ROP of CL at different temperatures under CH. Condition: [CL]:[FeCl_3_]=400:1


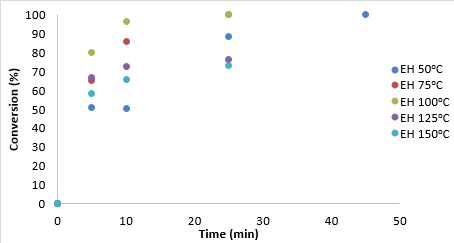


Figure S8 Conversion versus time plots for ROP of CL at different temperatures under EH. Condition: [CL]:[FeCl_3_]=400:1 at 150 W input power.


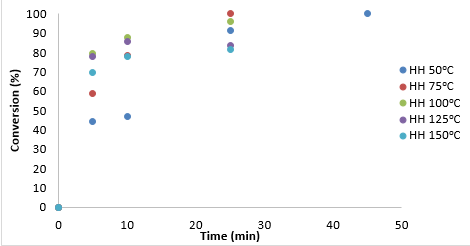


Figure S9 Conversion versus time plots for ROP of CL at different temperatures under HH. Condition: [CL]:[FeCl_3_]=400:1 at 150 W input power.

**Table S2 Average results of ROP of CL with FeCl_3_ and BzOH at various temperature at [M]:[C] ratio of 800:1**

| Entry | Temperature (^o^C) | Time (min) | Heating method | M_n_^a^  (g mol^-1^) | M_p_^a^  (g mol^-1^) | Ð ^a^ | conversion^b^ (%) |
| --- | --- | --- | --- | --- | --- | --- | --- |
| 1 | 50 | 120 | CH | 5000 | 7000 | 1.14 | 42.5 |
| 2 | 50 | 120 | EH | 6100 | 8400 | 1.15 | 68.8 |
| 3 | 50 | 120 | HH | 6400 | 8600 | 1.15 | 67.0 |
| 4 | 75 | 80 | CH | 5800 | 9400 | 1.42 | 61.0 |
| 5 | 75 | 80 | EH | 6600 | 9200 | 1.38 | 78.3 |
| 6 | 75 | 80 | HH | 7000 | 10000 | 1.31 | 74.3 |
| 7 | 100 | 45 | CH | 5000 | 7900 | 1.56 | 63.6 |
| 8 | 100 | 45 | EH | 5700 | 9800 | 1.46 | 76.4 |
| 9 | 100 | 45 | HH | 6300 | 11000 | 1.44 | 79.1 |
| 10 | 125 | 45 | CH | 3500 | 7100 | 1.66 | 51.1 |
| 11 | 125 | 45 | EH | 5200 | 8500 | 1.47 | 72.1 |
| 12 | 125 | 45 | HH | 5500 | 9700 | 1.51 | 70.2 |
| 13 | 150 | 60 | CH | 3100 | 6100 | 1.67 | 46.1 |
| 14 | 150 | 60 | EH | 4900 | 7700 | 1.54 | 71.2 |
| 15 | 150 | 60 | HH | 5300 | 9000 | 1.53 | 69.4 |

^a^ determined by GPC, measured in THF at 35^o^C

^b^ determined by ^1^H-NMR

**Table S3 Average results of ROP of CL with FeCl_3_ and BzOH at various temperature at [M]:[C] ratio of 1600:1**

| Entry | Temperature (^o^C) | Time (min) | Heating method | M_n_^a^  (g mol^-1^) | M_p_^a^  (g mol^-1^) | Ð ^a^ | Conversion^b^ (%) |
| --- | --- | --- | --- | --- | --- | --- | --- |
| 1 | 50 | 180 | CH | 3400 | 3700 | 1.06 | 28.6 |
| 2 | 50 | 180 | EH | 6900 | 7200 | 1.07 | 52.8 |
| 3 | 50 | 180 | HH | 6300 | 6700 | 1.11 | 47.5 |
| 4 | 75 | 180 | CH | 7000 | 7200 | 1.06 | 40.8 |
| 5 | 75 | 180 | EH | 9000 | 9400 | 1.12 | 69.5 |
| 6 | 75 | 180 | HH | 8800 | 9800 | 1.13 | 65.4 |
| 7 | 100 | 120 | CH | 6700 | 8900 | 1.23 | 51.7 |
| 8 | 100 | 120 | EH | 7500 | 10100 | 1.20 | 71.1 |
| 9 | 100 | 120 | HH | 7500 | 9700 | 1.24 | 67.9 |
| 10 | 125 | 120 | CH | 5700 | 8600 | 1.29 | 52.4 |
| 11 | 125 | 120 | EH | 7600 | 11000 | 1.22 | 70.8 |
| 12 | 125 | 120 | HH | 7300 | 11200 | 1.26 | 67.9 |
| 13 | 150 | 90 | CH | 5000 | 7300 | 1.30 | 48.2 |
| 14 | 150 | 90 | EH | 6900 | 10400 | 1.35 | 70.2 |
| 15 | 150 | 90 | HH | 6600 | 10000 | 1.31 | 62.8 |

^a^ determined by GPC, measured in THF at 35^o^C

^b^ determined by ^1^H-NMR


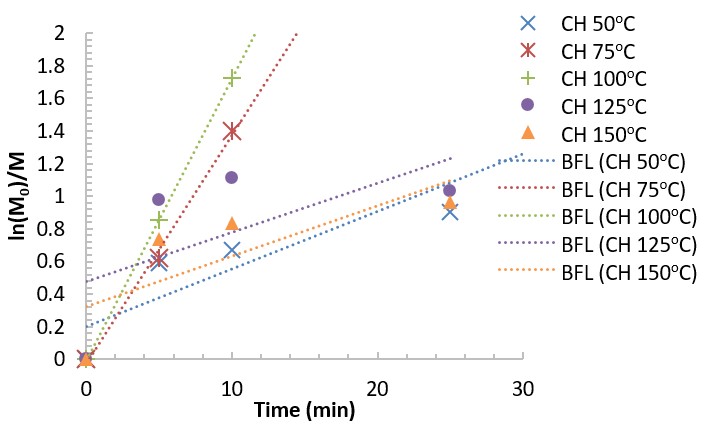


Figure S10 Kinetic plots of ln(Mo/M) versus time at different temperatures using the CH with best fit line (BFL). Condition: [CL]:[FeCl_3_]=400:1


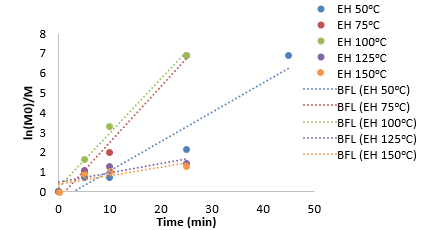


Figure S11 Kinetic plots of ln(Mo/M) versus time at different temperature using EH with best fit line (BFL). Condition: [CL]:[FeCl_3_]=400:1 at 150 W input power.


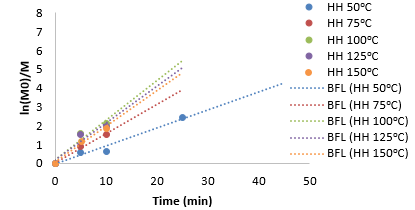


Figure S12 Kinetic plots of ln(Mo/M) versus time at different temperature using HH with best fit line (BFL). Condition: [CL]:[FeCl_3_]=400:1 at 150 W input power.


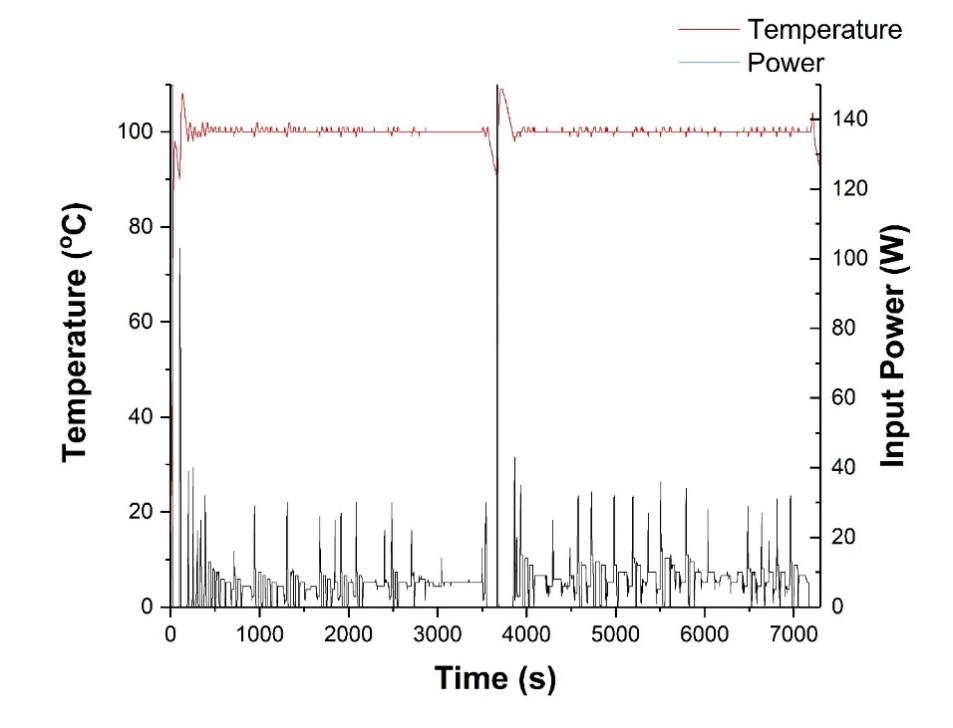


Figure S13 A typical temperature profile for ROP of CL with FeCl_3_ and BzOH at 100^o^C with EH.

**References:**

[1] J.L. Pedreño-Molina, J. Monzó-Cabrera, A. Lozano-Guerrero, and A. Toledo-Moreo, Design and Validation of a Ten-Port Waveguide Reflectometer Sensor: Application to Efficiency Measurement and Optimization of Microwave-Heating Ovens. Sensors 8 (2008) 7833-7849.
